# Genetic Analysis of *EGLN1* C127S Variant in Taiwanese Parkinson's Disease

**DOI:** 10.1155/2020/9582317

**Published:** 2020-04-25

**Authors:** Han-Lin Chiang, Chiung Mei Chen, Yi-Chun Chen, Chih-Ying Chao, Yih-Ru Wu, Guey-Jen Lee-Chen

**Affiliations:** ^1^Department of Neurology, Neurological Institute, Taipei Veterans General Hospital, Taipei 112, Taiwan; ^2^Department of Neurology, Chang Gung Memorial Hospital, Linkuo Medical Center, Chang Gung University College of Medicine, Taoyuan 333, Taiwan; ^3^Department of Neurology, Chang Gung Memorial Hospital, Linkuo Medical Center, Taoyuan 333, Taiwan; ^4^Department of Life Science, National Taiwan Normal University, Taipei 116, Taiwan

## Abstract

Parkinson's disease (PD) is a neurodegenerative disorder related to nigrostriatal dopaminergic neuron degeneration and iron accumulation. As a cellular oxygen sensor, prolyl hydroxylase domain containing protein 2 (PHD2, encoded by egl-9 family hypoxia inducible factor 1, *EGLN1*) modifies hypoxia-inducible factor alpha (HIF-*α*) protein for proteasomal destruction under normoxic condition. In addition, 2-oxoglutarate- (OG-) dependent dioxygenase activity of PHD2 is involved in the oxygen and iron regulation of iron-responsive element binding protein 2 (IRP2) stability. Previously increased expression of *EGLN1* was found in the substantia nigra of the parkinsonian brain. We investigated the possible role of c.380 G > C (p.C127S) of *EGLN1* gene in Taiwanese patients with PD. 479 patients and 435 healthy controls were recruited. Polymerase chain reaction and *Bsm*AI restriction enzyme analysis were applied for analysis. An association between CC genotype and reduced PD risk in the recessive model (CC vs. GG + GC) was found. Our study provides a link between *EGLN1* c.380 G > C SNP and the development of PD.

## 1. Introduction

Parkinson's disease (PD) is a neurodegenerative disorder characterized by *α-*synuclein aggregation in the brain and loss of dopaminergic neurons in the substantia nigra (SN). Although the disease etiology remains to be clarified, proteins encoded by PD-causing genes such as *SNCA, Parkin, DJ1, PINK1, and LRRK2* are involved in the oxidative stress pathway that contributes to neurodegeneration of PD [1]. Among the sources and mechanisms for the generation of oxidative stress, dysfunction of iron homeostasis and iron accumulation in the SN may be involved in the development of PD [2]. Previous study by Grunblatt et al. found increased gene expression of *EGLN1* (egl-9 family hypoxia inducible factor 1) in the SN of parkinsonian brains using oligonucleotide microarray technique [3]. The *EGLN1* gene encodes proline hydroxylase domain 2 (PHD2), which is an oxygen/iron sensor that belongs to the 2-oxoglutarate- (2-OG-) dependent dioxygenase superfamily. The upregulation of *EGLN1* promotes the degradation of hypoxic-inducible factor (HIF), an iron homeostasis-related protein [4]. Furthermore, evidence has shown that the iron regulatory protein 2 (IRP2), another key iron regulatory protein in mammals, was stabilized by a 2-OG-dependent dioxygenase specific inhibitor, dimethyloxalyglycine [5, 6], suggesting that *EGLN1* may downregulate IRP2. Intriguingly, in IRP2 knock-out mice, significant iron accumulation was found in the brain with neurodegeneration and adult onset ataxia, tremor, and bradykinesia [7]. Moreover, a high frequency of linked c.[12C > G; 380 G >C] or p.[Asp4Glu; Cys127Ser] variation in Tibetans [8] and Sherpas [9] was found in recent studies, which results in an adaptive function of the *EGLN1* for high altitude [8], although the finding was not replicated in the study of highland Andreans [10]. Thus, adaptation mechanisms involving *EGLN1* may or may not be similar among different populations. In this study, we investigated the possible role of c.380G > C (p.C127S, rs12097901) of the *EGLN* gene in Taiwanese patients with PD.

## 2. Materials and Methods

### 2.1. Ethics Statement

This study was performed under a protocol approved by the institutional review boards of Chang Gung Memorial Hospital (ethical license no: 95-0531B), and all examinations were performed after obtaining written informed consent.

### 2.2. Study Population

Patients diagnosed with PD were recruited from the neurology clinic of Chang-Gung Memorial Hospital. The diagnosis of PD was based on the UK PD Society Brain Bank clinical diagnostic criteria [11]. Unrelated healthy adult volunteers matched for age, gender, ethnic origin, and area of residences were recruited as controls.

### 2.3. Genetic Analysis

DNA was extracted from leukocytes by using the standard protocols. *EGLN1* gene polymorphism rs12097901 (c.380G > C, a Cys-to-Ser variant at position 127) was determined using the polymerase chain reaction and *Bsm*AI restriction enzyme analysis. Sequences of primers and polymorphic change in the enzyme recognition site were as follows: 5′-CCACACCAGCATTCCGGC-3′ (forward primer), 5′-TTGTTCATGCACGGCACGAT-3′ (reverse primer), and GTcTC (*Bsm*AI). The lowercase letter in the *Bsm*AI recognition site indicates the polymorphic site. The PCR was carried out in a reaction containing 100 ng genomic DNA, 0.2 *μ*M of each primer, 0.2 mM dNTPs, 1.5 mM MgCl_2_, 0.5 U *Taq* polymerase, and 10% DMSO. Thermal cycling conditions consisted of 94°C for 6 minutes for initial denaturation, followed by 35 cycles of 94°C for 30 seconds, 52°C for 30 seconds, and 72°C for 30 seconds. A final extension step of 72°C for 10 minutes was followed by a 4°C hold cycle. The amplified 401 bp PCR fragments, consisting of 75.3% CG content, were digested with the *Bsm*AI (New England Biolabs) and separated on a 2.0% agarose gel (allele G, 401-bp fragment; allele C, 233- and 168-bp fragments).

### 2.4. Statistical Analysis

The chi-square test was used to compare the frequency of the allele and genotypes in both cases and controls. Logistic regression analysis was performed to verify the interaction between age, sex, and the risk of PD. The genotypes of the subjects followed the Hardy–Weinberg equilibrium.

## 3. Results

We recruited 914 subjects, including 479 patients with PD (45.4% females) and 435 healthy controls (48.3% female). Only one proband with familial PD in the same family was recruited. The mean age at onset (AAO) of PD symptoms was 62.9 ± 10.7 years (range: 19–86), and the mean age of recruitment of 435 controls was 59.2 ± 12.7 years (range: 24–89). The frequencies of *EGLN1* c.380 G > C (p.C127S) alleles were similar in both PD patients and controls. However, the CC genotype was notably lower in the PD patient group (*p* = 0.056), and the results of the recessive model applied in genotype analysis (CC versus GG + GC) showed an association between CC and reduced PD risk (OR = 0.59, *p* = 0.002) (Table 1). Stratification by age at onset (<50 and ≧ 50 years) and sex also did not show differences in the minor C allele frequency in each case control cohort (data not shown).

## 4. Discussion

**Figure 1:**
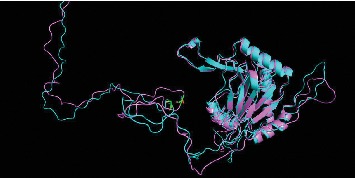
3D structure of proline hydroxylase domain 2 (PHD2). Wild type is labelled as blue, and variant is labelled as pink. The polymorphism sites are indicated by green bars.

The development of idiopathic PD is considered to be related with a complicated interplay of genetic and environmental factors affecting numerous fundamental cellular processes [12]. Among the environmental factors involved, hypoxic challenge can trigger neuronal cell death to exacerbate disease progression [13]. In addition, dysfunction in brain iron metabolism, specifically, iron accumulation in the SN, has been implicated in the pathogenesis of PD through increasing oxidative stress, impairing the ubiquitin proteasome system, and *α*-synuclein aggregation [2]. In this study, we studied the polymorphism of rs12097901 (c.380 G > C; p.C127S) of *EGLN1* gene in patients with PD and healthy controls. We found an association between CC and reduced PD risk in the recessive model of gene analysis (CC versus GG + GC). The role of this genetic variation in PD is still unknown but may be associated with HIF-related pathways. The product of *EGLN1* gene, PHD2, contains 426 residues, with the conserved catalytic domain located in the C-terminal region (∼181–426) [14]. The upregulation of EGLN1 promotes the degradation of HIF, and 2-OG-dependent-dioxygenases inhibitor stabilized IRP2 [5, 6]. We used protein structure prediction server [15] to confirm that this variant (p.C127S) did not change the protein structure at 3D level (Figure 1). Additionally, the pathogenicity of p.C127S was presumed to be benign by using SIFT and PolyPhen predictors. Although we did not check if this variant affects the gene expression level, kinetic analysis and functional experiments have shown that p.C127S variant has a lower Michaelis constant (Km) value for cosubstrate oxygen, suggesting that it promotes HIF degradation under hypoxic environment, hence blunting the hypoxic response in the literature [8]. The functional relevance of p.C127S (rs2790859) (an intergenic SNP downstream of EGLN1) alleles in an acute hypobaric hypoxia experiment was indicated as this variant was associated with percutaneous arterial oxygen saturation variations (SpO_2_) and SpO_2_ latency in a Japanese cohort [16]. Furthermore, loss of DJ-1, an autosomal recessive gene for familial PD, was shown to provoke hypoxic condition and increase reactive oxygen species production in human neuroblastoma cell, which in turn stabilized the HIF-1α. The posttranscriptional stabilization of HIF-1α was required to downregulate one of the receptors for the glial cell line-derived neurotrophic factor, ret proto-oncogene (RET). RET is strongly expressed in the dopaminergic (DA) neurons, and loss of RET expression in DA neurons generates PD-like dysfunctions in mouse models, thus indicating its crucial role in mesencephalic DA cell survival. Since RET is prosurvival, this study implicates that increased HIF-1α-associated response may contribute to neurodegeneration in PD [17].

IRP2 is a posttranscriptional regulatory protein that binds to the iron-responsive element (IREs) in the mRNA of various iron regulatory proteins to facilitate iron uptake and prohibit iron storage during an iron deficient state [18]. Studies have suggested that 2-OG-dependent dioxygenase inhibitor and hypoxia or hypoxia mimetics stabilize IRP2 [5, 6], and N-terminally truncated form of PHD2 (residues 181-426) exhibits high affinity for, and copurifies with the endogenous level of, its Fe^2+^ cofactor and 2-oxoglutarate cosubstrate [19], linking 2-OG-dependent dioxygenase activity to IRP2 degradation. Therefore, based on our study result, it is speculated that 2-OG-dependent dioxygenase activity of PHD2 may be influenced by p.C127S to affect brain IRP2 and alters iron metabolism, contributing to the observed reduced PD risk. However, whether p.C127S affects the PHD2 activity is not known. Future studies in the functional consequence of p.C127S on the 2-OG-dependent dioxygenase activity and IRP2 degradation are necessary to further clarify how p.C127S affects PD risk. In conclusion, our study provides evidence that the protective C127S variant of *EGLN1* is associated with reduced risk of PD. The limitation of our study is that only one SNP in *EGLN1* was analyzed and that future studies analyzing other possible genetic variations should be warranted for further investigation.

## Figures and Tables

**Table 1:** Distributions and association of *EGLN1* p.C127S (TGT > TCT) polymorphism in patients and controls.

	PD (%)	NC (%)	OR (95% CI)	*p*
	(*n* = 479)	(*n* = 435)		
G	56.6	53.2	1.00	
C	43.4	46.8	0.87 (0.72–1.05)	0.150
GG	28.8	30.3	1.00	
GC	55.5	45.7	1.27 (0.95–1.72)	0.110
CC	15.7	23.9	0.69 (0.47–1.01)	0.056

Recessive model				

GC + GG	84.3	76.1	1.00	
CC	15.7	23.9	0.59 (0.42–0.82)	0.002

## Data Availability

The datasets used and/or analyzed during the current study are available from the corresponding author upon reasonable request.
